# Microbiological Quality and Analysis of *Escherichia coli* Resistance to Antibiotics in Beaches: Evaluating Anthropic Influence in Surface Waters in Brazil

**DOI:** 10.1002/wer.70645

**Published:** 2026-10-29

**Authors:** Monica M. Elias, Francini dos R. Henrique, Philipe R. Gomes, Daniele G. dos Santos, Roberta R. Rangel, Layanne A. Mendonça, Eliane C. de Vasconcelos, Maurício Dziedzic, Cíntia Mara R. de Oliveira

**Affiliations:** ^1^ Graduate Program in Environmental Management (PPGAMB), Universidade Positivo Curitiba Brazil; ^2^ Federal Institute of Education, Science and Technology Fluminense – IFFluminense Campus Itaboraí Itaboraí Brazil; ^3^ PPG Ecology and Natural Resources North Fluminense State University Darcy Ribeiro Campos dos Goytacazes Brazil; ^4^ Federal Institute of Education, Science and Technology Fluminense – IFFluminense Campus Campos Guarus Campos dos Goytacazes Brazil; ^5^ Federal Institute of Education, Science and Technology Fluminense – IFFluminense Campus Campos Centro Campos dos Goytacazes Brazil; ^6^ School of Engineering University of Northern British Columbia Prince George British Columbia Canada

**Keywords:** antibiotic resistance, estuary pollution, multiresistant bacteria, public health, recreational water

## Abstract

Antimicrobial resistance is a problem for public health. We sought to assess the multiple antibiotic resistance analyses by searching for resistant microorganisms in five Brazilian beaches. The analyses showed pH (5.2–8.5), temperature (21.2°C–27.5°C), and dissolved oxygen (DO) (4.0–8.5 mg L^−1^). Bacterial indicators peaked during the high season, notably at Beach 5 (4.60 × 10^2^ ± 1.71 × 10^2^ MPN.100 mL^−1^), although Beaches 1 (*p* 0.056) and 2 (*p* 0.097) showed significant seasonal coliform variation (*p* < 0.10). Overall, 83.3% of isolated *Escherichia coli* strains were resistant to at least one antibiotic, mainly strains resistant to imipenem (45.0%) and cefazolin (35.0%). Multivariate analysis explained 86.70% of the variance, which resulted in high antibiotic resistance index‐IRAp (e.g., IRAp_Beach5_ 0.818, IRAp_Beach1_ 0.273) and multiple antibiotic resistance (MAR_Beach2_ = 0.60). Significant anthropogenic pressure‐driven by untreated wastewater, proximity to the continent, ports, industrial complexes, and overpopulation contributed to the proliferation of resistant microorganisms, whereas rainfall leakage contributed to resilience in the area.

## Summary

- Anthropogenic activities drive coastal water contamination in Sepetiba Bay.
- Lack of wastewater treatment is a major source of microbial pollution.
- High prevalence of antibiotic‐resistant *E. coli* was observed (83.3%).
- Multidrug‐resistant strains (up to 12 antibiotics) were identified.
- Tourism and proximity to mainland and industrial areas increase contamination levels.

## Graphical abstract

Anthropic activities and untreated wastewater promote the dissemination of antibiotic‐resistant *E. coli* (83.3% of isolates) in coastal waters, increasing environmental contamination and public health risks.

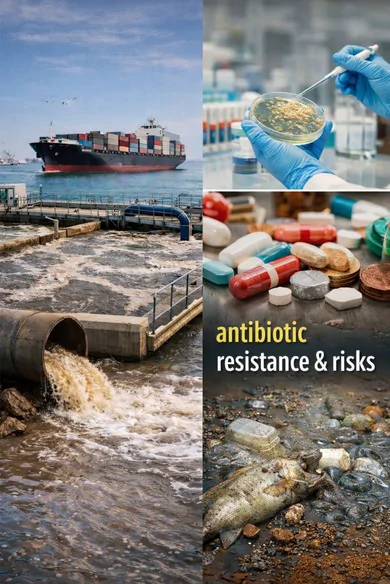

## 1 Introduction

The intensive use of antimicrobials coupled with the inadequate disposal of fresh wastewater and effluents from both water resource recovery facility (WRRF) and effluent of hospitals, has disseminated antimicrobial resistance (AMR) in the aquatic environment (Souza et al. 2009; Guerra et al. 2020; Alibi et al. 2021; Cailleau et al. 2024; Mondal et al. 2024; Chen et al. 2025; Ringenary et al. 2026). These waters may contain a high concentration of chemical pollutants, such as metals and antimicrobials alongside resistant organisms and genes (Döhla et al. 2020; Moreira et al. 2021; da Silva et al. 2022; Wang et al. 2022; Oliveira et al. 2024; Chen et al. 2025; Izma et al. 2025; Kim and Lee 2025; Reis et al. 2025; Pittaccio et al. 2026; Rodrigues Júnior et al. 2026). It is known that fecal pollutants and antibiotic resistance genes (ARGs) may promote enhanced resistance to metals, with implications for bacterial colonization and transmission in the healthcare environment (Moreira et al. 2021; Reddy et al. 2022; Wang et al. 2022; Izma et al. 2025; McGee et al. 2026; Pittaccio et al. 2026).

Currently, resistant organisms can be found in humans, animals, food, and the environment (Webster et al. 2004; Souza et al. 2009; Ragugnetti et al. 2011; Silva et al. 2011; Vasconcelos et al. 2017; Jeong et al. 2021; Mello et al. 2022; Montiel‐Mora et al. 2026). They expand the possibility of spreading these organisms, selecting new resistant individuals (Döhla et al. 2020; Ringenary et al. 2026).

The dissemination of these organisms in the environment occurs through multiple factors, either through horizontal transfer of resistant genes, through mutations, or through the migration of these genes to other regions through the aquatic environment (Colet et al. 2021; Oliveira et al. 2024; Thangrongthong et al. 2025; Pittaccio et al. 2026; Sastre‐Dominguez et al. 2026). This fact made these impacts a public health problem (Maloo et al. 2017; Nadimpalli et al. 2018; Zhang et al. 2020; Ringenary et al. 2026). The movement of people also facilitated the spread of these organisms, because gram‐negative bacteria are easily transferred by the intestinal flora. Among these bacteria, *Escherichia coli* , a thermotolerant coliform, indicates a significant influence of fecal contamination in the environment. It possesses a highly fluid genome mediated by plasmids, which disseminate resistance to antibiotics in the environment (Millan et al. 2016; Sastre‐Dominguez et al. 2026), favoring the use of these organisms as indicators of fecal contamination (Silva et al. 2011; Ghaderpour et al. 2015; Maloo et al. 2017; Zhang et al. 2020; Jeong et al. 2021).

Multiple antibiotic resistance (MAR) has been observed in several environments, mainly in low‐income countries. Rodrigues Júnior et al. (2026) found that 70% of strains isolated in coastal lagoon, Maranhão (Brazil), were resistant; Isaiah et al. (2025) observed 76.9% resistant *E. coli* in Port Harcourt Metropolis (Nigeria), from which 42% exhibited resistance to at least four antibiotics. Costa et al. (2026) detected frequent resistance across multiple antibiotic classes, resulting in high MAR to 10 antibiotics (MAR = 0.62) in Guanabara Bay, RJ, Brazil. In contrast Döhla et al. (2020), in Germany, observed only 5% of the samples with resistant strains in recreational waters.

To minimize the spread of these types of organisms, the World Health Organization has established strategies and priorities that aim to stimulate the conscious use of antimicrobials, reduce the incidence of infections, and develop policies that minimize the dispersion of resistant organisms and genes (Nadimpalli et al. 2018; WHO – World Health Organization 2023). Together with these actions, it was recommended that investments be made in the public health area, such as the construction of WRRFs (Cailleau et al. 2024; Sikder et al. 2024) and achieve universal and equitable access to safe and affordable drinking water for all (United Nations (UN) 2022).

Sepetiba Bay (SB) routinely receives fresh domestic wastewater, and even so, only one study was found that evaluated these contaminants in the region, in which the authors estimated coliform of density values of up to two orders of magnitude, including on Beach 5, object of study of this paper (Copeland et al. 2003; INEA 2026). Regarding antibiotic resistance in SB, there are two papers using AMR: The first evaluated the occurrence of pharmaceuticals in seafood (Mello et al. 2022), and the second investigated AMR genes obtained from sediments (Moreira et al. 2022). It is known that pollutants dispersed on the continental shore of SB are carried for up to 5 km inland from the bay (Rodrigues et al. 2009), a dynamic that can influence on the water quality and dispersion resistance genes from/to Atlantic Ocean (Moreira et al. 2022; Damasceno et al. 2024). In this sense, pharmaceuticals are considered important environmental biomarkers and have been the subject of studies (Kim and Lee 2025; Rodrigues Júnior et al. 2026).

The present study aimed to assess the dispersion of resistant genes in the environment of Itacuruçá Island, carried out by evaluating the microbiological quality and determining the resistance index of *E. coli* to antibiotics in five beaches, which have distinct levels of occupation and environmental impacts.

## 2 Methodology

This research was authorized by the ethics committee, under Certificate of Appreciation number 58103716.5.0000.0093 and opinion 1,732,348, in compliance with Resolution N° 196/96 by the Ministry of Health.

### 2.1 Area Study and Specimen Collection

SB is in the southeast of the state of Rio de Janeiro (RJ) (Figure 1), in a region of high economic value and great real estate expansion, being explored by the tourism industry and port and industrial complexes (Figure 1B) (Oliveira 2015; Euzebio 2024). This set of factors combined with unbalanced regional growth led to the disposal of wastewater (Copeland et al. 2003) and in natural marine and industrial effluents at SB (Pinto et al. 2019; Carvalho et al. 2022; Reis et al. 2025). These reduced environmental quality in the region, even interfering in the well‐being and health of the population and of the local fauna and flora (Afonso et al. 2011; Araújo et al. 2017; da Silva et al. 2022; Euzebio 2024).

**FIGURE 1:**
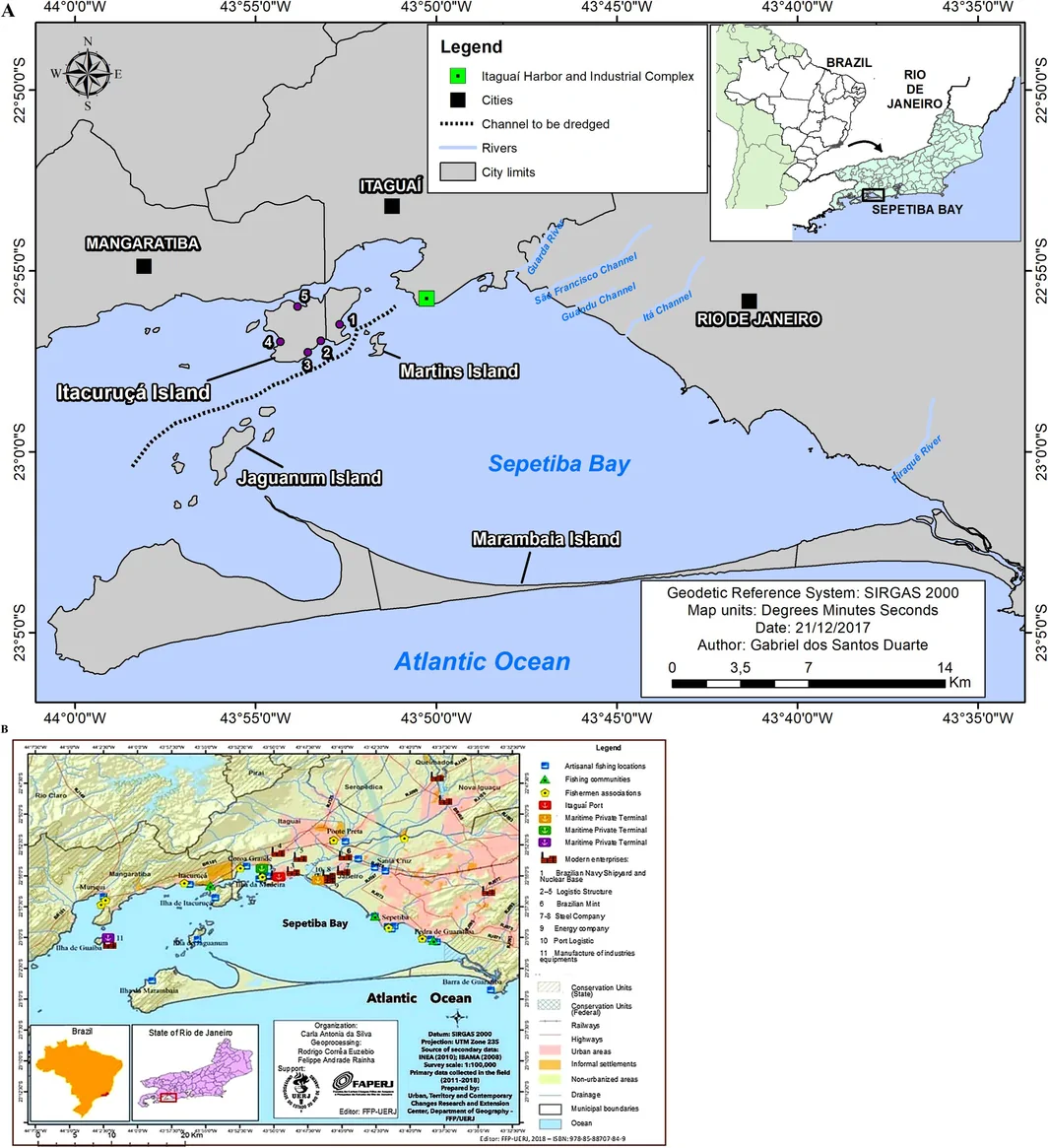
Study of the area. (A) Sepetiba Bay (SB, Rio de Janeiro, Brazil). Emphasis on the beaches of Itacuruçá Island, sampling sites named: 1 = Quatiquara, strong impacts from dredgings, ports and industries; 2 = Águas Lindas, impacts from the dredging channel; 3 = Maria Russa, low anthropogenic influence; 4 = Praia Grande, most influenced by the Atlantic Ocean; 5 = Gamboa, the highest anthropogenic pressure, due to its residents and the influence of the pollutants from the continent (Mangaratiba and Itaguaí cities). *Source:* Gabriel dos S. Duarte. (B) Main activities in Sepetiba Bay. Adapted from Euzebio (2024).

Itacuruçá Island (IT; Figure 1A) is the second largest in extension among the SB islands, with 900 ha. It does not receive drinking water from the continent, and the water consumed is taken from the springs. Wastewater is dispensed in gulches, as there are no treatment plants in the region (Vianna 1998; SNIS 2022). It is the island with the highest population density, and it receives the largest number of tourists and vacationers from December to February and July. The resident population lives mainly from fishing and tourism (Euzebio 2024).

IT has 16 small beaches, among which, for this research, are the ones with the highest population density on weekends. The chosen ones were Quatiquara (Beach 1, latitude: 22°56′28.7″ S; longitude: 043°52′40.4″ W); Águas Lindas (Beach 2, latitude: 22°56′55.8″ S; longitude: 043°53′11.7″ W); Praia Grande (Beach 4, latitude: 22°56′57.5″ S; longitude: 043°54′19.0″ W); Gamboa (Beach 5, latitude: 22°55′59.1″ S; longitude: 43°53′50.3″ W). In addition, Maria Russa was considered as low anthropic influence reference (Beach 3, latitude: 22°57′15.2″ S; longitude: 043°53′33.0″ W), because there are two houses and only one inhabitant.

The number of residents was determined by asking questions directly to residents and the number of houses (both residents and vacationers) by counting the houses one by one.

Surface water samples were collected in sterile borosilicate glass bottles, kept refrigerated, and immediately transported to the laboratory (APHA 2023). Five samples were collected monthly, between November 2015 and November 2016, except in April 2016 (Table 1), yielding 60 samples for coliform analysis; however, antibiotic resistance testing was performed on only 55 of these samples, as December 2015 was excluded due to technical issues. Sampling spanned 12 months, covering both rainy (September to January) and dry (February to August) periods.

**TABLE 1:** Values of the tide height at the time of collection (Tides 2016) and the sum of precipitation in the 10 days prior to collection (INMET 2016).

Season classification^a^	Samples	Tide (cm)	Precipitation (mm)	Month classification according to weather season^b^
High	1–5	30	59	Rainy
	6–10	30	100	
	11–15	140	40	
	16–20	140	1	Dry
	21–25	150	1	
Low	26–30	150	1	
	31–35	20	8	
High (school vacation)	36–40	30	1	
	41–45	90	1	
Low	46–50	90	32	Rainy
	51–55	120	27	
	56–60	220	8	

^a^ High season is the period of greatest attendance of tourists and vacationers.

^b^ The meteorological classification was based on the volume of rain in the ten days prior to collection.

### 2.2 Water's Physical and Chemical Properties

Analyses performed in situ were water temperature (in°C) and dissolved oxygen (DO) (in mg L^−1^), and DO and temperature quantifications were performed using the portable dissolved oxygen meter Lutron DO‐5519. In the laboratory, they measured the pH of the samples; pH quantifications were performed using the pH meter (Gekara, PG1800, São Paulo, Brazil).

### 2.3 Microbiological Tests

The measurement of total coliforms (TC) and thermotolerant coliforms (TTC) was performed using the multiple tube fermentation technique done in quintuplicate (APHA 2023). The samples were diluted in saline solution (0.9% w/v) in the concentrations: 10^−1^, 10^−2^, 10^−3^, 10^−4^, and 10^−5^ (in g L^−1^).

In the identification of *E. coli* strains, positive tubes were sown on methylene blue eosin agar (Kasvi), and those with bluish black color with reflections in metallic green were inoculated on MacConkey agar (Himedia) and on agar nutrient (Himedia). The slabs were kept for 48 h at 35°C (Drying and Sterilization Oven LT 501, LimaTec Laboratory Equipment, Bahia, Brazil).

When the plates containing MacConkey agar showed red or pink coloration, the corresponding plates in nutrient agar were subjected to the biochemical test for enterobacterales, using the commercial test kit (Newprov) (Cavalieri et al. 2005). The positive colonies in the *E. coli* characterization test were subjected to antibiotic resistance testing.

The resistance of *E. coli* strains to antibiotics was verified using the disk diffusion technique for the following antibiotics: amikacin 30 μg (AMI), ampicillin 10 μg (AMP), ampicillin + sulbactam 20 μg (ASB), aztreonam 30 μg (ATM), cefazolin 30 μg (CFZ), cefepime 30 μg (CPM), cefotaxime 30 μg (CTX), cefoxitin 30 μg (CFO), ceftazidine 30 μg (CAZ), ceftriaxone 30 μg (CRO), ciprofloxacin 5 μg (CIP), chloramphenicol 25 μg (CHL), cotrimoxazole 25 μg (SUT), gentamicin 10 μg (GEN), imipenem 10 μg (IPM), levofloxacin 5 μg (LVX), meropenem 10 μg (MER), nitrofurantoin 300 μg (NIT), piperacillin + tazobactam 110 μg (PPT), and tetracycline 30 μg (TET). For this purpose, *E. coli* colonies grown on nutrient agar were diluted in saline solution until a turbidity corresponding to 0.5 on the McFarland scale was obtained and seeded in petri dishes with Mueller–Hinton agar (Himedia), where they were distributed in five or six disks impregnated with antibiotics, placed in equidistant spaces and incubated for 24 h at 35°C. Then, the diameters of the inhibition halos were measured (CLSI 2023).

To verify the resistance of microorganisms, indexes were determined whose results can be related to the impact generated by each antibiotic (Webster et al. 2004; Isaiah et al. 2025; Costa et al. 2026) in relation to the others, as described in Equation (1).

(1) $$\mathrm{IRA}=\frac{\text{number of antibiotics to which the isolate was resistant}}{\text{total number of antibiotics tested}}$$

where IRA means strain resistance index to antibiotic.

For the determination of the resistance index by collection site (Equation 2), the number of resistance tests was used in relation to the total tests performed per beach (Webster et al. 2004).

(2) $$\text{IRAp}=\frac{\text{number of tests with positive results for resistance in each collection site}}{\text{total number of tests}\;\mathrm{per}\;\text{collection site}}$$

where IRAp means the strain resistance index to antibiotics per collection site.

For the determination of the resistance index by collection site in relation to strains isolated (Equation 3), the number of antibiotics resistant was used in relation to tests with positive results for resistance in each collection site (Costa et al. 2026).

(3) $$\mathrm{MAR}=\frac{\text{total number of antibiotics to which the isolate was resistant in each collection site}}{\text{number of the isolates with resistance to collection site}\times \text{total number of antibiotics tested}}$$

where MAR means multiple resistance index per collection site.

### 2.4 Statistical Treatment

The CT variable was subjected to the *t*‐test, and those that did not have a normal distribution were subjected to the Wilcoxon‐Mann–Whitney test when comparing the effects of the weather season and the season. The SPSS software, Version 30.0, for Windows, was used to perform the multivariate analysis, where the microbiological variables and physical properties were standardized with zero mean and standard deviation in order to be evaluated by the factor analysis technique by the extraction method of main components with equal scale (Hair et al. 2019; Pallant 2020).

The extraction of the number of factors followed the Guttman–Kaiser criterion for the selection of the components that presented eigenvalues greater than one (1.0) (Floyd and Widaman 1995; Coppers et al. 2021). In this sense, the variables whose exclusion generated a higher Cronbach alpha compared to the one presented, considering all the initial study variables, or which had an item‐total correlation below 0.50, were removed from the analysis (Hair et al. 2019). In a second step, the values related to the rainy and dry seasons and high and low seasons were tested using the *t*‐Student test to compare two or more samples from a set of parametric data and the Wilcoxon‐Mann–Whitney test for nonparametric data, adopting a 90% confidence level (Pallant 2020).

Hydrometeorological variables (tide height and precipitation in the last 10 days prior to collection; Tables 1 and S1) were related to physico‐chemical properties (pH, DO, and T; Tables 2 and S2–S4), occupation of each beach (Table 4), and microbiological variables (TC and TTC; Tables 3, S5, and S6). The number of antibiotics to which strains were resistant per beach (nARB; Tables 5 and S7–S11) and number of strains that were resistant to antibiotics on each beach (nSR; Table 5).

**TABLE 2:** The height of the tide at the time of collection (Tides 2016), precipitation in the 10 days prior to collection (INMET 2016), average dissolved oxygen (average DO), temperature (T), and average pH of the five beaches.

Samples	Tide (cm)	Precipitation (mm)	Average DO (mg L^−1^)	Average T (°C)	Average pH
1–5	30	59	—	—	5.7
6–10	30	100	—	—	—
11–15	140	40	7.0	26.8	8.4
16–20	140	1	5.4	25.7	8.1
21–25	150	1	5.6	26.8	7.7
26–30	150	1	6.0	23.7	8.0
31–35	20	8	—	—	—
36–40	30	1	—	—	7.9
41–45	90	1	—	—	8.2
46–50	90	32	5.3	23.8	7.6
51–55	120	27	5.2	23.5	8.3
56–60	220	8	5.5	23.7	—
Average	100	23	5.7	24.9	7.8
Standard deviation	63.5	30.9	0.6	1.5	0.8

*Note:* “—” indicates samples in which the analysis was not performed.

**TABLE 3:** Comparison of the averages or posts of the amount of TTC for each beach in the dry and rainy seasons and in the high and low seasons.

Beach	Thermotolerant coliforms (TTC) (MPN.100 mL^−1^)					
	Dry season	Rainy season	*p*	High season	Low season	*p*
1	2.21 × 10^2^ ± 2.36 × 10^2^	6.80 × 10^1^ ± 1.32 × 10^2^	0.056*	2.21 × 10^2^ ± 2.36 × 10^2^	6.80 × 10^1^ ± 1.32 × 10^2^	0.136
2	2.34 × 10^2^ ± 4.32 × 10^2^	4.75 × 10^1^ ± 5.12 × 10^1^	0.175	2.68 × 10^2^ ± 4.14 × 10^2^	1.42 × 10^1^ ± 1.80 × 10^1^	0.097*
3	6.67 ± 1.03 × 10^1^	1.97 × 10^1^ ± 3.28 × 10^1^	0.116	1.97 × 10^1^ ± 3.02 × 10^1^	6.67 ± 1.63 × 10^1^	0.198
4	3.17	3.33	0.930	3.58	2.91	0.558
5	2.27 × 10^2^ ± 1.71 × 10^2^	1.28 × 10^2^ ± 1.77 × 10^2^	0.212	1.98 × 10^2^ ± 1.73 × 10^2^	1.58 × 10^2^ ± 1.89 × 10^2^	0.377

*Note:* The values are presented as means ± standard deviation and *p*‐value of Student's *t*‐test for Beaches 1, 2, 3, and 5. Beach 4 did not present normal data, and, therefore, the stations and the *p*‐value of the Wilcoxon–Mann–Whitney test were performed, adopting a 90% confidence level.

Abbreviation: MPN, most probable number.

* Significant difference was observed between seasons or weather seasons with *p* < 0.10.

## 3 Results and Discussion

### 3.1 Physical–Chemical Parameters and Environmental Variables

The pH found in the present study varied between 5.2 ± 0.5 and 8.5 ± 0.1 (Table 2), mean of 7.8 ± 0.8. However, specifically only after the holiday, the observed pH average 5.7 ± 0.5 (Table S2). This value was below the limit established by the current national environmental legislation in force for the good quality of recreational water (pH: 6.5–8.5) (CONAMA 2005). Similarly, Damasceno et al. (2024) and de Freitas et al. (2019) reported pH values of 7.78–8.74 and 6.5–8.5 in SB, respectively, which also aligns with the findings of Alibi et al. (2021) in an estuary in Tunisia.

The water temperature varied according to the seasons (21.2°C ± 2.3°C–27.5°C ± 0.6°C, Tables 2 and S3), similarly to Damasceno et al. (2024) in SB, reported T values of 25.1°C–25.6°C.

DO varied between 4.9 ± 0.5 and 8.5 ± 1.0 mg L^−1^ (Tables 2 and S4). DO values below the ideal by the current national environmental legislation (6 mg L^−1^, CONAMA 2005) were observed in 65.7% of these samples, suggesting contributions from high number of tourists, environmental pollutants, by the drought in the dry season (Table S4) or dredging (Damasceno et al. 2024; Reis et al. 2025). de Freitas et al. (2019) observed in SB a little temperature variation (23.7°C–24.1°C) and (6.5–7.0 mg L^−1^) for DO, both in the same season.

According to the statistical analysis, the DO values in 1 and 5 were lower than ideal, probably due to the greater pollution of these beaches, which can influence environmental health, deteriorating water quality, generating unpleasant odors and flavors, and causing risks to the health of the users (Döhla et al. 2020; Damasceno et al. 2024; Reis et al. 2025).

### 3.2 Total Coliforms (TC), Thermotolerants (TTC), and Occupation

Fecal indicator bacteria such as total coliforms were detected in 61.7% of samples, whereas *E. coli* was isolated in 32.7% of strains (Table 3). Concentrations ranged from < 1.8 to 1.7.10^3^ MPN.100 mL^−1^ for TC (Table S5) and from < 1.8 to 1.3.10^3^ MPN.100 mL^−1^ for TTC (Table S6). Thermotolerant coliform concentrations exceeded the permissible limit in dry season (Table S6) during vacation month, in July and after holiday in November (CONAMA 2000, 2005). Ringenary et al. (2026) observed that coliform indicators (TC or TTC) exceeded bathing thresholds 60.3% of the time, based on an analyzed during 28 years.

It was observed that the values were directly influenced by occupation (number of residents, vacationers, and tourist frequency; Table 4) and precipitation some days prior to collection (Table S6). The total number of residents in the IT was estimated at 932 people, with Beach 5 being the most populated (Table 4).

**TABLE 4:** Occupation (number of residents and vacationers' houses) at the studied beaches. Swimmability is determined for each beach based on the TTC values of five consecutive months (CONAMA 2000, 2005).

Occupation and swimmability	Beach					Total
	1	2	3	4	5	
Residents' houses	20	20	1	25	300	366
Number of residents	40	35	1	56	800	932
Vacationers' houses	80	100	1	120	50	351
Total of houses	100	120	2	145	350	717
Swimmability between November15 and March 16 (CONAMA 2000, 2005)	Very good	Excellent	Excellent	Very good	Very good	
Swimmability between May 16 and September 16 (CONAMA 2000, 2005)	Very good	Excellent	Excellent	Excellent	Excellent	

There was no significant difference in the density of Beach 5 coliforms (Table 3) between the seasons, probably because it was mostly occupied by residents (Table 4).

The swimmability of the beaches was classified as “excellent” in the low season, reaching “very good” at the time of greatest occupation (Tables 4 and S6) despite the recurrent increase in the discharge of raw wastewater at that time, both on the island and on the mainland (Copeland et al. 2003). In high season, high levels of coliforms were observed on beaches 2 and 5 (Table 3), with values reaching even above those allowed by current legislation (CONAMA 2000, 2005) indicating very poor water quality and highest degree of environmental disturbance in holidays during dry season. Uddin et al. (2023) also observed the highest *E. coli* value in urban water bodies in Dhaka, Bangladesh.

During visits to Itacuruçá Island, the local population complained about unpleasant odors on Beaches 1 and 5 and were uncomfortable stating that this made it impossible to use the beach for recreation. This fact corroborates the results of the analysis of the main components (Figure 2), which indicated that Beaches 1 and 5 were the most polluted, both in relation to microbiological activity (Table 3) and due to the low DO value (Table 2).

**FIGURE 2:**
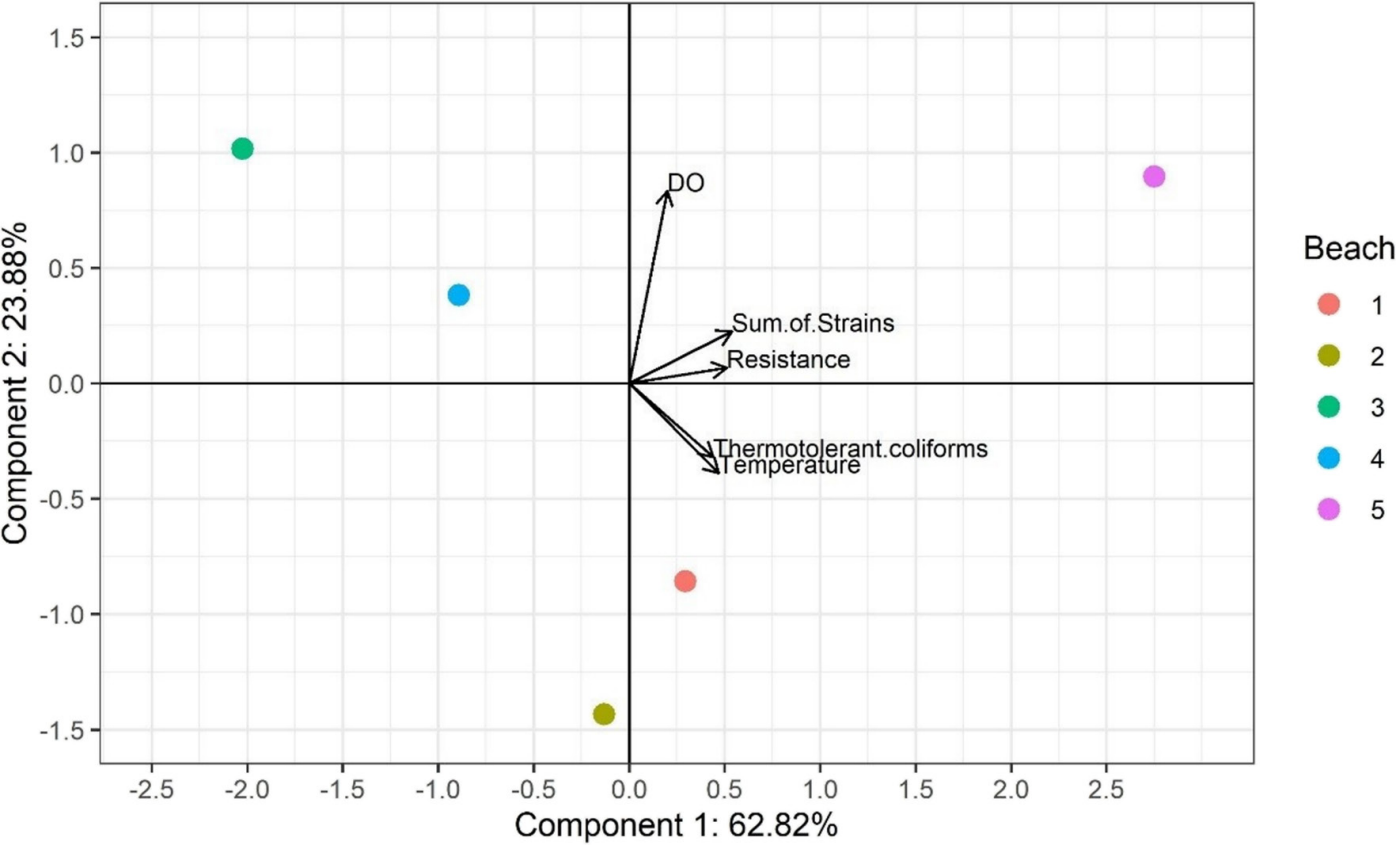
Biplot of the main components relating to the most significant variables and the beaches obtained through analysis of the main component (ACP): nSR, nARB, T, and TTC.

According to the tests of comparison of means and stations, only Beach 1 had a significant difference in TTC (*p* < 0.10) between the dry and rainy seasons (Table 3). Beach 2 showed a significant difference in TTC (*p* < 0.10) between high and low season (Table 3), where antibiotic‐resistant bacteria were found only in high season, which coincided with the dry season, characterizing greater contamination in this period.

Beaches 3 and 4, located farther from the continent, had less microbial load. Although Beach 4 receives more vacationers (Table 4), no influence of this factor was observed in TTC (Table 3), showing that this environment was able to absorb the impacts even during high season. As it faces the ocean (Figure 1), probably Beach 4 is less influenced by pollution from the continent, in addition to the circulation of water bodies from the ocean that disperse pollutants (Amado Filho et al. 2003; Rodrigues et al. 2009; Moreira et al. 2022) diluting the microbial load.

Analyses carried out by the State Environment Institute (INEA) classified several beaches around the SB as inappropriate (INEA 2026). The justification for this is that the cities that surround the bay have a wastewater collection network, but the collected wastewater is not treated (SNIS 2022) being dispensed in water bodies (Copeland et al. 2003; IBGE 2017; SNIS 2022), probably reaching to SB (Pinto et al. 2019; Moreira et al. 2022).

Pinto et al. (2019) also found that proximity to the continent (Figure 1) influenced microbial load as well as Copeland et al. (2003) who measured the concentration of coliforms on the Mangaratiba Shore and found a difference of up to two orders of magnitude between it and Beach 5. Jeong et al. (2021) also noted that the dilution of pollutants, especially in the rainy season, was able to reduce the density of coliforms.

The measured values for TTC made it possible to observe the direct influence of occupation, without treating effluents, on the microbial load of the beaches. In Beaches 1 and 2, a high microbial load was observed in high season (Table 4) and a sharp reduction with the decrease in the number of vacationers; Beach 3 had TTC values of 2.0 × 10^1^ MPN.100 mL^−1^, until, in October, a trailer was installed that sold snacks and drinks, causing a greater occupation of the beach. In the following month, the increase in coliform density to 4.0 × 10^1^ MPN.100 mL^−1^ and the presence of resistant strains was already observed (Table 5).

**TABLE 5:** Resistance profile of *E. coli* strains to one or more antibiotics among 20 tested antibiotics.

No. of antibiotics to which the strains were resistant	Antibiotics	Beach					Number of drug classes (WHO 2023)
		1 (*n* = 4)	2 (*n* = 2)	3 (*n* = 1)	4 (*n* = 1)	5 (*n* = 10)	
0	—	1	1	—	—	1	—
1	CFZ	—	—	—	—	1	
	CHL	1	—	—	—	—	
	IPM	1	—	1	—	3	—
	GEN	—	—	—	—	—	
	CHL	1	—	—	—	—	
2	IPM–TET	1	—	—	—	—	2
3	AMP–CFZ–TET	—	—	—	1	—	3
	CFZ–CPM–CAZ	—	—	—	—	1	1
	CFZ–CTX–CHL	—	—	—	—	1	2
	CFZ–CFO–IPM	—	—	—	—	1	2
9	AMP–ASB–ATM–CFZ–CPM–CTX–CAZ–CRO–CHL	—	—	—	—	1	4
12	AMP–ASB–ATM–CFZ–CPM–CTX–CFO–CAZ–CRO–CHL–IPM–LVX	—	1	—	—	—	6
No. of resistant strains ≥ 1 antibiotic		3 (75%)^a^	1 (50%)^a^	1 (100%)^a^	1 (100%)^a^	9 (90%)^a^	
No. of resistant strains ≥ 2 antibiotics		1 (25%)^a^	1 (50%)^a^	0	1 (100%)^a^	5 (50%)^a^	
No. of resistant strains ≥ 3 antibiotics		0	1 (50%)^a^	0	1 (100%)^c^	5 (50%)^a^	
No. of resistant strains ≥ 4 antibiotics		0	1 (50%)^a^	0	0	1 (10%)^a^	
Strains no thermotolerant in 12 samples (*n* = 11)		7	9	10	10	1	
Beach resistance index (IRAp)		0.273	0.091	0.091	0.091	0.818	
MAR index		0.067	0.600	0.050	0.150	0.133	

*Note:* Beaches: Quatiquara (1); Águas Lindas (2); Maria Russa (3); Praia Grande (4); Gamboa (5); n, number of strains found per beach; —, no resistance was observed.

^a^ Percentage of resistant strains in relation to the total strains found on that beach (n). Tested antibiotics: amikacin (AMI), ampicillin (AMP), ampicillin + sulbactam (ASB), aztreonam (ATM), cefazolin (CFZ), cefepime (CPM), cefotaxime (CTX), cefoxitin (CFO), cefoxitin (CFO), ceftazidine (CAZ), ceftriaxone (CRO), ciprofloxacin (CIP), chloramphenicol (CHL), cotrimoxazole (SUT), gentamicin (GEN), imipenem (IPM), levofloxacin (LVX), meropenem (MER), nitrofuran (NIT), piperacillin + tazobactam (PPT), polymyxin B (POL), and tetracycline (TET).

The results observed in the present paper corroborate the observations in the literature on the direct influence of the concentration of coliforms and the presence of bacteria resistant to antibiotics and metallic pollutants (da Silva et al. 2022; Mello et al. 2022; Moreira et al. 2022; Damasceno et al. 2024; Chen et al. 2025; Reis et al. 2025; Rodrigues Júnior et al. 2026) due to the dispersion of metallic contaminants and untreated wastewater or with inadequate treatment to remove micropollutants (Guerra et al. 2020; Jeong et al. 2021; Costa et al. 2026). Based on the above, it is known that actions aimed at the reduction dispersion of metallic contamination and construction of WRRFs could reduce environmental degradation in SB, as observed by Costa et al. (2026) and Cailleau et al. (2024). Wastewater treatment eliminated over 98% of resistant strains across all evaluated targets (Cailleau et al. 2024). Consequently, ecosystem restoration may yield significant intangible economic, social, and environmental benefits.

### 3.3 *E. coli* Resistance to Tested Antibiotics

The absence of wastewater treatment is a global public health problem, and it is estimated that 42% of global wastewater production is dispensed in untreated environments (UNICEF 2025). SB and adjacent systems illustrate the impacts of chronic pollution by metallurgical and wastewater, exhibiting high levels of fecal and heavy metal contamination. In this scenario, we evaluated *E. coli* resistance against 20 antibiotics (across 10 antimicrobial classes) that are widely prescribed and consumed by the population, which are part of the basic list of drugs provided by the Brazilian Federal Government (Brazil 2024).

Of 55 samples, 18 (32.7%) had positive results for *E. coli* (Table 5 and Figure S1B). Among these, 15 isolates showed resistance to at least one of the tested antibiotics (83.3%), whereas 44% exhibited multiple resistance (to at least two antibiotics).

Antibiotic resistance can be observed against one antibiotic (AMR) or multiple classes of antibiotics (MAR). This is particularly concerning, as some strains demonstrate resistance to at least three classes of antibiotics (Isaiah et al. 2025). The present investigation evaluated resistance across 10 antimicrobial classes (Table S12), demonstrating that 33% of these strains multidrug resistance (MDR). Specifically, 6% of isolates were resistant to 12 antibiotics on Beach 2 (6 drug classes), 6% to 9 antibiotics on Beach 5 (4 drug classes), and 22% to 3 antibiotics (3 drug classes on Beach 4, whereas 1 and 2 drug classes on Beach 5) (Table 5). The results demonstrated an elevated risk to ecosystems, especially on Beaches 2 and 5. MAR was observed at all sites except Beach 3, where a single strain displayed resistance to imipenem following the November after holyday weekend. Costa et al. (2026) found that 12 strains MAR isolated from Guanabara Bay, RJ, Brazil, exhibited resistance to three or more antibiotics. Alibi et al. (2021) observed that 90% of strains isolated were multiple resistant to antibiotics.

Although the resistance indexes were calculated at all beaches, two beaches outperformed the others in the number of resistant strains, on Beaches 1 (Index IRAp = 0.273; Table S7) and 5 (Index IRAp = 0.818; Table S11) (Table 5). Resistance index values higher than 0.2 indicate high‐risk sources of environmental contamination, as already reported by Costa et al. (2026) and Isaiah et al. (2025). Regarding major pollution sites, Beaches 1 and 5 demonstrated an elevated risk to the ecosystem, along with environmental degradation occurrence of elevated number of *E. coli* and multiple resistant bacteria. On Beach 5, bathers cited cases of sick children after bathing in the sea, which corroborates Afonso et al. (2011) who evaluated data from nearby health centers and found a high risk of developing gastroenteric diseases when performing recreational activities in the region. Similar research was carried out with children who suffered diarrhea in 50% of which the presence of *E. coli* was confirmed (Zil‐E‐Huma et al. 2019).

A high MAR index was recorded at Beach 2 (MAR = 0.600), where one strain was resistant to six classes of antibiotics (Tables 5 and S8), the highest resistance level observed, which elevated risk to the ecosystem and intense environmental degradation, probably caused by the high population in holiday weekend, dredging, absence of wastewater treatment, and proximity to the continent. Costa et al. (2026) observed a maximum MAR index of 0.62, where 85% of strains were categorized as high risk (MAR > 0.2) in Guanabara Bay, Brazil.

Three antibiotics displayed high IRA Index values, reaching maximum resistance to imipenem (IRA = 0.45), cefazolin (IRA = 0.35), and chloramphenicol (IRA = 0.20) (Tables 5 and S13 and Figure S1). Strains resistant to imipenem were found in four of the five beaches, suggesting that people or hospital wastewater in the region has been dispensed at SB, reinforcing the need to build WWTPs in the region and in hospitals around the SB (Mello et al. 2022). Thus, these effluents are of great impact for water bodies, being a public health issue (Guerra et al. 2020; Mello et al. 2022; Cailleau et al. 2024).

Antibiotic resistance (AMR) has been observed in several environments (Rodrigues Júnior et al. 2026). The strains isolated in a coastal lagoon in Maranhão, Brazil, were resistant cephalothin (70%), tetracycline (41%), and chloramphenicol (73%), in contrast to Döhla et al.'s (2020) analyses in Germany, which observed only 5% of the samples with resistant strains. In the Kampala, Uganda, within the dry season, over 65% of the locations presented a high risk for antibiotic resistance (Twinomucunguzi et al. 2022). Colet et al. (2021) observed *E. coli* in artesian wells from Brazil, 100% grown in norfloxacin and 77.8% in gentamicin.

The second antibiotic with the highest number of resistant strains (35%) was cefazolin, found on three beaches (2, 4, and 5; Table 5). This antimicrobial was classified as having a high environmental risk due to its frequent consumption, which could lead to an increase in diversity and the number of ARGs (Souza et al. 2009). Research in a children's hospital showed an average level of risk quotient for this antibiotic (Guerra et al. 2020).

In Beaches 1, 2, and 5, resistant strains with resistance to chloramphenicol were verified (IRA 0.20). Nevertheless, Mello et al. (2022) did not detect chloramphenicol in seafood samples in SB. Alibi et al. (2021) observed that 80% of strains isolated from an estuary in Tunisia were resistant to chloramphenicol. In four beaches, resistant strains or intermediate strains to ampicillin were verified, with resistance index of IRA 0.15. The remaining antibiotic exhibited IRA < 0.15, and all strains were sensitive to amikacin, ciprofloxacin, cotrimoxazole, gentamicin, meropenem, nitrofurantoin, and piperacillin + tazobactam.

The highest levels of resistance were observed in March and November, after the holiday weekend, a time of great visitation by tourists and vacationers and the absence of precipitation (Table 1) in the days preceding the collection and wastewater treatment plants, suggesting that these factors impacted the microbial load (Table 5). Other factors that can raise the observed rates are the normal hydrodynamic movements of the bay, such as tides and currents, as well as the dredging processes, which can raise the observed rates even further. Because the microorganisms disposed in the estuarine bed can resuspend with dredging, increasing the concentration of *E. coli* in the water column by up to 20 times (Saibro et al. 2023).

The nine variables observed in the present study were grouped into two factors in the execution of the multivariate analysis and analyzed according to a model capable of explaining 86.70% of the data variances. In the evaluation of principal component analysis (ACP; Figure 2 and Tables S14 and S15), according to the Guttman–Kaiser criterion, two main components were extracted, where:

- 62.82% was explained by Component 1, which corresponded to the variability of the microbiological activity of the water, with the most important variables being sum of strains, resistance, T, and TTC;
- 23.88% for Component 2, which corresponded to the variation of the physical attribute, consisting only of the variable DO. The results again suggest that Beach 1 and especially Beach 2 have the lowest concentrations of dissolved oxygen. This suggests that variations in resistance are largely independent of DO concentrations.

In Beaches 1 and 5, the largest numbers of resistant strains were observed, confirming the need for actions to treat wastewater, to reduce the observed IRAp index (0.818; Table 5), IRA index (0.45 to imipenem and 0.35 to cefazolin), and MAR index (0.60 for beach 2), suggesting high environmental risk. In addition to these impacts, metallic pollutants contribute to the findings of Moreira et al. (2023), which reported high ecological risk in metal polluted environments within SB, where it is estimated that more than 70% of microbial taxa are affected.

The other beaches are more distant from the continent and showed a smaller number of resistant strains (Table 5). In the analysis of main components (Figure 2), these beaches appear in the opposite direction to the growth of microbiological activity, and, therefore, they can be considered less polluted. However, among them, Beach 2 deserves considerable attention because it is closer to the sense of increased microbiological activity. This can be an indication of high environmental risk, especially when considering the high value of IRAp and MAR index that was observed in high season (Table 5) and the proximity to the dredging channel in Beaches 1–3 (Figure 1), contributing to the reduction of the resilience capacity of this environment (Carvalho et al. 2022; Saibro et al. 2023).

It was observed that wastewater from residences on the Itacuruçá Island, together with fecal pollution from the continent, influenced the rates of multiple resistance indexes, especially in dry months when there was a large circulation of people on the beaches. The environment still has a resilience capacity, but it is essential to build wastewater treatment plants throughout the SB region and the islands to favor the maintenance of this environment's health. Fecal contamination of recreational seawater represents a real risk to ecosystem, human health, and public health (Alibi et al. 2021).

In recent years, SB has experienced several spillages of metallic pollutants (Pinto et al. 2019; Damasceno et al. 2024; Reis et al. 2025). Research has shown that when genes resistant to metals are found in environments with domestic pollutants, they can present a positive correlation with genes resistant to antibiotics and favor the increase in the number of these genes in the environment (Zhang et al. 2020; Reddy et al. 2022; Wang et al. 2022; Oliveira et al. 2024; Chen et al. 2025). Despite the absence of such research in SB, it is known that the high rates of the two classes of contaminants can favor the multiple resistance of microorganisms to metals and drugs together (Zhang et al. 2020; Chen et al. 2025; Rodrigues Júnior et al. 2026). In addition, dredging processes can resuspend microorganisms favoring MDR simultaneously to the two classes of pollutants (Moreira et al. 2021; Saibro et al. 2023).

Urban water resources are becoming significant reservoirs of ARGs and hotspots for resistant microorganisms as an emerging hotspot of AMR, contributing to the dissemination of these organisms (Cailleau et al. 2024). Estuaries in areas of high urbanization and industrialization, such as SB (Figure 1B), characterize significant pollution due to massive discharges of anthropogenic waste containing antibiotics, pesticides, heavy metals, and antibiotic‐resistance genes (Alibi et al. 2021; Gripp et al. 2022; Moreira et al. 2022; Paiva et al. 2025; Ringenary et al. 2026). The increasing prevalence of these ARGs threatens the effective treatment of bacterial infections (Pittaccio et al. 2026).

Strategies are essential for enhancing public health in environments that are more and more affected by pollution and anthropogenic activities. Kim and Lee (2025) demonstrated that improved biosecurity practices further reduced antimicrobial usage and index of the *E. coli* resistance in Broiler Farms in Korea.

The study has contributed with information for city officials and policymakers to understand the pollution levels and sources in SB. The findings hold the potential to facilitate the formulation of imperative measures aimed at counterbalancing the negative ramifications of pollution on both the bay and its surrounding community. It is recommended that the local government provide restrictive policies for the treatment and assessment of municipal wastewater before its discharge into sea.

## 4 Conclusion

The lack of treatment of pollutants discharged in water bodies that flow into SB has caused negative impacts, both in the bay and on the beaches of Itacuruçá Island, in the southeast region of the state of Rio de Janeiro, Brazil. The microbiological analysis provided data that prove the need for this treatment, given the high number of resistant and multiple resistance strains, indicating fecal contamination in the region's water resources, putting human and environmental health at risk. The results obtained can guide investment policies in fecal pollution remediation programs and confirm the need for action by the authorities in the studied region. Further studies can explore the mechanisms governing microbial interactions with antibiotic and metal resistance genes in SB. It is essential to develop goals aimed at protecting health and culminating in the elimination of the impacts that occur in the region, in addition to the implementation of policies aimed at promoting public health.

## Author Contributions

**Monica M. Elias:** conceptualization, investigation, funding acquisition, writing – original draft, methodology, validation, visualization, writing – review and editing, formal analysis, project administration, data curation, resources. **Francini dos R. Henrique:** formal analysis, data curation. **Philipe R. Gomes:** formal analysis, investigation. **Daniele G. dos Santos:** formal analysis, investigation. **Roberta R. Rangel:** investigation, formal analysis. **Layanne A. Mendonça:** formal analysis, data curation, validation. **Eliane C. de Vasconcelos:** conceptualization, supervision, project administration, funding acquisition, methodology, validation, writing – original draft. **Maurício Dziedzic:** data curation, writing – review and editing. **Cíntia Mara R. de Oliveira:** data curation, supervision, writing – original draft, writing – review and editing, validation.

## Funding

This work was supported by Universidade Positivo and Instituto Federal de Educação, Ciência e Tecnologia Fluminense, and the Article Processing Charge (APC) for the publication of this research was funded by the Coordenação de Aperfeiçoamento de Pessoal de Nível Superior ‐ Brasil (CAPES) (ROR identifier: 00x0ma614).

## Conflicts of Interest

The authors declare no conflicts of interest.

## Supporting information


**Table S1:** Classification about weather and season. Tide height at collection time and precipitation in the 10 days prior to collection (mm).
**Table S2:** pH. Data are presented for each sampling point (Beach 1–Beach 5) comparing the rainy and dry seasons.
**Table S3:** Temperature (T) (°C). Data are presented for each sampling point (Beach 1–Beach 5) comparing the rainy and dry seasons.
**Table S4:** Dissolved oxygen (DO) (mg L^−1^). Data are presented for each sampling point (beach 1–beach 5) comparing the rainy and dry seasons.
**Table S5:** Total coliforms (TC) (MPN.100 mL^−1^). Data are presented for each sampling point (Beach 1–Beach 5) comparing the rainy and dry seasons.
**Table S6:** Thermotolerant coliforms (TTC) (MPN.100 mL^−1^). Data are presented for each sampling point (Beach 1–Beach 5) comparing the rainy and dry seasons. Swimmability is determined for each beach based on the TTC values of 5 consecutive months (CONAMA 2000, 2005).
**Table S7:** Resistance profile of *E. coli* strains to one or more antibiotics among 20 tested antibiotics on the Beach 1. Index IRA = resistance index to antibiotic, Index IRAp = resistance index to antibiotics per beach, Index MAR = multiple antibiotic resistance per beach.
**Table S8:** Resistance profile of *E. coli* strains to one or more antibiotics among 20 tested antibiotics on the Beach 2. Index IRA = resistance index to antibiotic, Index IRAp = resistance index to antibiotics per beach, Index MAR = multiple antibiotic resistance per beach.
**Table S9:** Resistance profile of *E. coli* strains to one or more antibiotics among 20 tested antibiotics on the Beach 3. Index IRA = resistance index to antibiotic, Index IRAp = resistance index to antibiotics per beach, Index MAR = multiple antibiotic resistance per beach.
**Table S10:** Resistance profile of *E. coli* strains to one or more antibiotics among 20 tested antibiotics on the Beach 4. Index IRA = resistance index to antibiotic, Index IRAp = resistance index to antibiotics per beach, Index MAR = multiple antibiotic resistance per beach.
**Table S11:** Resistance profile of *E. coli* strains to one or more antibiotics among 20 tested antibiotics on Beach 5. Index IRA (resistance index to antibiotic), Index IRAp (resistance index to antibiotics per beach), Index MAR (multiple antibiotic resistance per beach).
**Table S12:** The presented antimicrobials are grouped into the following pharmacological classes (WHO 2023).
**Table S13:** Antimicrobial susceptibility profile of *E. coli* –positive samples.
**Figure S1:** (A) Comparison of antibiotic resistance among *Escherichia coli* isolates from some of the beaches. (B) Antimicrobial class resistance among *E. coli* isolates from each beach.
**Table S14:** Factor coordinates of the variables, based on correlations.
**Table S15:** Eigenvalues of correlation matrix and related statistics. Active variables only.

## Data Availability

The data that support the findings of this study are available as Supporting Information.
