# Supplementary material for: Microbiological Quality and Analysis of Escherichia coli Resistance to Antibiotics in Beaches: Evaluating Anthropic Influence in Surface Waters in Brazil
Source: Water Environ Res. 2026 Oct 29;98(11):e70645. doi: 10.1002/wer.70645 (PMC13719888; doi:10.1002/wer.70645)
Supplement: Supplementary file 1 [file WER-98-e70645-s001.docx]

**Supplementary Materials**

**Microbiological quality and analysis of *Escherichia coli* resistance to antibiotics in beaches: evaluating anthropic influence in surface waters in Brazil**

# **Table S1**

Table S1. Classification about weather and season. Tide height at collection time and precipitation in the ten days prior to collection (mm).

| **MONTH** | 15-NOV | 15-DEC | 16-JAN | 16-FEB | 16-MAR | 16-MAY | 16-JUN | 16-JUL | 16-AUG | 16-SEP | 16-OCT | 16-NOV |
| --- | --- | --- | --- | --- | --- | --- | --- | --- | --- | --- | --- | --- |
| **Samples** | 1-5 | 6-10 | 11-15 | 16-20 | 21-25 | 26-30 | 31-35 | 36-40 | 41-45 | 46-50 | 51-55 | 56-60 |
| Precipitation in the ten days prior to collection (mm) | 59 | 100 | 40 | 1 | 1 | 1 | 8 | 1 | 1 | 32 | 27 | 28 |
| Information about weather | rainy rainy rainy | | | dry dry dry dry dry dry | | | | | | rainy rainy rainy | | |
|  |  | | |  | | | | | |  | | |
| Height of the tide at the time of collection (cm) | 30 | 30 | 140 | 140 | 150 | 150 | 20 | 30 | 90 | 90 | 120 | 220 |

# **Table S2**

Table S2. pH. Data are presented for each sampling point (beach 1 – beach 5) comparing the rainy and dry seasons.

| **MONTH** | **15-NOV** | **15-DEC** | **16-JAN** | **16-FEB** | **16-MAR** | **16-MAY** | **16-JUN** | **16-JUL** | **16-AUG** | **16-SEP** | **16-OCT** | **16-NOV** | **Average** | **Standard Deviation** |
| --- | --- | --- | --- | --- | --- | --- | --- | --- | --- | --- | --- | --- | --- | --- |
| **Samples** | 1-5 | 6-10 | 11-15 | 16-20 | 21-25 | 26-30 | 31-35 | 36-40 | 41-45 | 46-50 | 51-55 | 56-60 |  |  |
| Beach 1 | 5.2 | - | 8.5 | 8.1 | 7.4 | 8.0 | - | 7.7 | 8.1 | 7.4 | 8.2 | - | 7.6 | 1.0 |
| Beach 2 | 5.4 | - | 8.3 | 8.1 | 7.7 | 8.1 | - | 7.9 | 8.3 | 7.7 | 8.4 | - | 7.8 | 0.9 |
| Beach 3 | 5.5 | - | 8.4 | 8.1 | 7.8 | 8.0 | - | 8.0 | 8.2 | 7.7 | 8.3 | - | 7.8 | 0.9 |
| Beach 4 | 6.2 | - | 8.4 | 8.1 | 7.8 | 8.0 | - | 8.0 | 8.3 | 7.7 | 8.3 | - | 7.9 | 0.7 |
| Beach 5 | 6.2 | - | 8.5 | 8.1 | 7.8 | 8.0 | - | 8.0 | 8.3 | 7.7 | 8.4 | - | 7.9 | 0.7 |
| **Average** | 5.7 | - | 8.4 | 8.1 | 7.7 | 8.0 | - | 7.9 | 8.2 | 7.6 | 8.3 | - |  |  |
| **Standard Deviation** | 0.5 | - | 0.1 | 0.0 | 0.2 | 0.0 | - | 0.1 | 0.1 | 0.2 | 0.1 | - |  |  |

- samples in which the analysis was not performed.

# **Table S3**

Table S3. Temperature (T) (°C). Data are presented for each sampling point (beach 1 – beach 5) comparing the rainy and dry seasons.

| **MONTH** | 15-NOV | 15-DEC | 16-JAN | 16-FEB | 16-MAR | 16-MAY | 16-JUN | 16-JUL | 16-AUG | 16-SEP | 16-OCT | 16-NOV | **Average** | **Standard Deviation** |
| --- | --- | --- | --- | --- | --- | --- | --- | --- | --- | --- | --- | --- | --- | --- |
| **Samples** | 1-5 | 6-10 | 11-15 | 16-20 | 21-25 | 26-30 | 31-35 | 36-40 | 41-45 | 46-50 | 51-55 | 56-60 |  |  |
| **Season** | rainy | rainy | rainy | dry | dry | dry | dry | dry | dry | rainy | rainy | rainy |  |  |
| Beach 1 | - | **-** | 27.5 | 25.9 | 27.0 | 25.8 | - | - | - | 24.0 | 23.7 | 23.8 | 25.4 | 1.6 |
| Beach 2 | - | **-** | 27.2 | 24.9 | 26.7 | 24.9 | - | - | - | 23.6 | 23.3 | 24.2 | 25.0 | 1.5 |
| Beach 3 | - | **-** | 26.0 | 25.2 | 26.8 | 21.2 | - | - | - | 23.7 | 23.4 | 23.4 | 24.2 | 1.9 |
| Beach 4 | - | **-** | 26.2 | 25.6 | 26.6 | 21.2 | - | - | - | 23.8 | 23.5 | 23.5 | 24.3 | 1.9 |
| Beach 5 | - | **-** | 27.0 | 26.8 | 27.1 | 25.4 | - | - | - | 23.7 | 23.7 | 23.7 | 25.3 | 1.6 |
| **Average** | - | - | 26.8 | 25.7 | 26.8 | 23.7 | - | - | - | 23.8 | 23.5 | 23.7 |  |  |
| **Standard Deviation** | - | - | 0.6 | 0.7 | 0.2 | 2.3 | - | - | - | 0.2 | 0.2 | 0.3 |  |  |

- samples in which the analysis was not performed.

# **Table S4**

Table S4. Dissolved oxigen (DO) (mg.L^-1^)*.* Data are presented for each sampling point (beach 1 – beach 5) comparing the rainy and dry seasons.

| **MONTH** | 15-NOV | 15-DEC | 16-JAN | 16-FEB | 16-MAR | 16-MAY | 16-JUN | 16-JUL | 16-AUG | 16-SEP | 16-OCT | 16-NOV | **Average** | **Standard Deviation** |
| --- | --- | --- | --- | --- | --- | --- | --- | --- | --- | --- | --- | --- | --- | --- |
| **Samples** | 1-5 | 6-10 | 11-15 | 16-20 | 21-25 | 26-30 | 31-35 | 36-40 | 41-45 | 46-50 | 51-55 | 56-60 |  |  |
| **Season** | rainy | rainy | rainy | dry | dry | dry | dry | dry | dry | rainy | rainy | rainy |  |  |
| Beach 1 | **-** | **-** | 6.8 | 5.4 | 6.0 | 6.3 | - | - | - | 4.8 | 4.0 | 5.7 | 5.6 | 0.9 |
| Beach 2 | - | - | 6.4 | 4.9 | 5.4 | 5.1 | - | - | - | 5.4 | 4.5 | 5.5 | 5.3 | 0.6 |
| Beach 3 | **-** | **-** | 7.1 | 5.0 | 5.2 | 6.2 | - | - | - | 5.4 | 6.1 | 5.3 | 5.8 | 0.7 |
| Beach 4 | - | - | 6.0 | 5.4 | 5.9 | 6.2 | - | - | - | 5.8 | 5.6 | 5.6 | 5.8 | 0.3 |
| Beach 5 | **-** | **-** | 8.5 | 6.1 | 5.3 | 6.0 | - | - | - | 4.9 | 5.6 | 5.6 | 6.0 | 1.2 |
| **Average** | - | - | 7.0 | 5.4 | 5.6 | 6.0 | - | - | - | 5.3 | 5.2 | 5.5 |  |  |
| **Standard Deviation** | - | - | 1.0 | 0.5 | 0.4 | 0.5 | - | - | - | 0.4 | 0.9 | 0.2 |  |  |
| - samples in which the analysis was not performed. | | | | | | | | | | | | |  |  |

# **Table S5**

Table S5. Total coliforms (TC) (MPN.100mL^-1^). Data are presented for each sampling point (beach 1 – beach 5) comparing the rainy and dry seasons.

| **MONTH** | **15-NOV** | **15-DEC** | **16-JAN** | **16-FEB** | **16-MAR** | **16-MAY** | **16-JUN** | **16-JUL** | **16-AUG** | **16-SEP** | **16-OCT** | **16-NOV** | **Average** | **min** | **max** | **Standard Deviation** |
| --- | --- | --- | --- | --- | --- | --- | --- | --- | --- | --- | --- | --- | --- | --- | --- | --- |
| **Samples** | 1-5 | 6-10 | 11-15 | 16-20 | 21-25 | 26-30 | 31-35 | 36-40 | 41-45 | 46-50 | 51-55 | 56-60 |  |  |  |  |
| **Season** | rainy | rainy | rainy | dry | dry | dry | dry | dry | dry | rainy | rainy | rainy |  |  |  |  |
| **Beach 1** | 68 | 260 | <1.8 | 490 | 1300 | 330 | 20 | 78 | 540 | 18 | <1.8 | 230 | 278 | <1.8 | 1300 | 374 |
| **Beach 2** | 20 | 110 | 210 | 130 | 220 | 20 | 20 | 1700 | 20 | 61 | 20 | 140 | 223 | 20 | 1700 | 471 |
| **Beach 3** | 45 | 220 | 20 | 20 | 45 | <1.8 | <1.8 | <1.8 | 20 | <1.8 | <1.8 | 40 | 34 | <1.8 | 220 | 61 |
| **Beach 4** | 1300 | 790 | <1.8 | 840 | 68 | <1.8 | 18 | <1.8 | 45 | 20 | <1.8 | 78 | 263 | <1.8 | 1300 | 447 |
| **Beach 5** | 1700 | 280 | <1.8 | 490 | 220 | 480 | 78 | 330 | 490 | 45 | 20 | 230 | 364 | <1.8 | 1700 | 458 |
| **Average** | 627 | 332 | 46 | 394 | 371 | 166 | 27 | 422 | 223 | 29 | 8 | 144 |  |  |  |  |
| **Standard Deviation** | 810 | 264 | 92 | 327 | 526 | 225 | 30 | 727 | 267 | 24 | 11 | 87 |  |  |  |  |

Abbreviation: MPN, most probable number.

# **Table S6**

Table S6. Thermotolerant coliforms (TTC) (MPN.100mL^-1^)	. Data are presented for each sampling point (beach 1 – beach 5) comparing the rainy and dry seasons. Swimmability is determined for each beach based on the TTC values of five consecutive months (CONAMA, 2005; 2000)

| MONTH | 15-NOV | 15-DEC | 16-JAN | 16-FEB | 16-MAR | 16-MAY | 16-JUN | 16-JUL | 16-AUG | 16-SEP | 16-OCT | 16-NOV | Average | min | max | Standard Deviation | |
| --- | --- | --- | --- | --- | --- | --- | --- | --- | --- | --- | --- | --- | --- | --- | --- | --- | --- |
| Samples | 1-5 | 6-10 | 11-15 | 16-20 | 21-25 | 26-30 | 31-35 | 36-40 | 41-45 | 46-50 | 51-55 | 56-60 |  |  |  |  |  |
| Season | rainy | rainy | rainy | dry | dry | dry | dry | dry | dry | rainy | rainy | rainy |  |  |  |  |  |
| Beach 1 | <1.8 | 330 | <1.8 | 45 | 410 | 330 | <1.8 | <1.8 | 540 | <1.8 | <1.8 | 78 | 144 | <1.8 | 540 | | 199 |
| Beach 2 | <1.8 | 110 | 110 | 45 | 220 | <1.8 | 20 | **1100** | 20 | <1.8 | 20 | 45 | 141 | <1.8 | 1100 | | 309 |
| Beach 3 | <1.8 | 78 | <1.8 | <1.8 | 20 | <1.8 | <1.8 | <1.8 | 20 | <1.8 | <1.8 | 40 | 13 | <1.8 | 78 | | 24 |
| Beach 4 | **1300** | 330 | <1.8 | 20 | 68 | <1.8 | 18 | <1.8 | 20 | <1.8 | <1.8 | 20 | 148 | <1.8 | 1300 | | 374 |
| Beach 5 | 460 | 200 | <1.8 | 45 | 490 | 330 | 45 | 230 | 220 | 45 | 20 | 45 | 178 | <1.8 | 490 | | 174 |
| Average | 352 | 210 | 22 | 31 | 242 | 132 | 17 | 266 | 164 | 9 | 8 | 46 |  |  |  |  | |
| Standard Deviation | 566 | 119 | 49 | 20 | 206 | 181 | 19 | 477 | 227 | 20 | 11 | 21 |  |  |  |  | |
| Swimmability | Beach 1 - Very Good  Beach 2 – Excellent  Beach 3 – Excellent  Beach 4 - Very Good  Beach 5 - Very Good | | | | | Beach 1 - Very Good  Beach 2 – Excellent  Beach 3 – Excellent  Beach 4 – Excellent  Beach 5 - Excellent | | | | |  |  |  |  |  |  | |

In Bold, thermotolerant coliform concentrations exceeded the permissible limit for class I waters, suitable for primary contact recreation, established by CONAMA Resolutions N° 274/2000 and N° 357/2005.

# **Table S7**

Table S7. Resistance profile of E. coli strains to one or more antibiotics among 20 tested antibiotics on the Beach 1. Index IRA (resistance index to antibiotic), Index IRAp (resistance index to antibiotics per beach), Index MAR (multiple antibiotic resistance per beach).

| **Samples** | **16-FEB** | | **16-MAY** | **16-AUG** | **16-NOV** | **Frequency R (n=4)** | **Frequency I (n=4)** | **Frequency S (n=4)** | **IRA = N° R/n (n=4)** |
| --- | --- | --- | --- | --- | --- | --- | --- | --- | --- |
|  | 16-20 | | 26-30 | 41-45 | 56-60 |  |  |  |  |
| **Tested antibiotics:** | dry | | dry | dry | rainy |  |  |  |  |
| AMI | S | | S | S | S | 0 | 0 | 4 | 0 |
| AMP | S | | **I** | S | S | 0 | 1 | 3 | 0 |
| ASB | S | | S | S | S | 0 | 0 | 4 | 0 |
| ATM | S | | S | S | S | 0 | 0 | 4 | 0 |
| CFZ | **I** | | S | **I** | S | 0 | 2 | 2 | 0 |
| CPM | S | | **I** | S | S | 0 | 1 | 3 | 0 |
| CTX | S | | S | S | S | 0 | 0 | 4 | 0 |
| CFO | S | | S | S | S | 0 | 0 | 4 | 0 |
| CAZ | S | | S | S | S | 0 | 0 | 4 | 0 |
| CRO | S | | S | S | S | 0 | 0 | 4 | 0 |
| CIP | S | | S | S | S | 0 | 0 | 4 | 0 |
| CHL | **R** | | **I** | S | S | 1 | 1 | 2 | 0.25 |
| SUT | S | | S | S | S | 0 | 0 | 4 | 0 |
| GEN | S | | S | S | S | 0 | 0 | 4 | 0 |
| IPM | S | | S | **R** | **R** | 2 | 0 | 2 | 0.5 |
| LVX | S | | S | S | S | 0 | 0 | 4 | 0 |
| MER | S | | S | S | S | 0 | 0 | 4 | 0 |
| NIT | S | | S | S | S | 0 | 0 | 4 | 0 |
| PPT | S | | **I** | S | S | 0 | 1 | 3 | 0 |
| TET | S | | S | S | **R** | 1 | 0 | 3 | 0.25 |
| **Antibiotic resistance per point (drug Class)** | **1 (1)** | | **0** | **1 (1)** | **2 (2)** | IRAp for beach = n with strain resistance  N° Samples  IRAp for beach = 3  11  **IRAp for beach** = **0.273** | | | |
| **Antibiotic intermediate per point** | **1** | | **4** | **1** | **0** |  |  |  |  |
| **IRAp for sample =** N° AMR/∑ antibiotics = ∑R/20 | **0.05** | | **0.00** | **0.05** | **0.10** |  |  |  |  |
| **Intermediate Index =** N° Antibiotic intermediate/ ∑ antibiotics = ∑I/20 | **0.05** | | **0.20** | **0.05** | **0.00** |  |  |  |  |
| **MAR Index** = N° AMR/(n with strain resistance x ∑ antibiotics) = ∑R /(3*20) | | **0.067** | | | |  | | | |

Analysis was not performed on samples 6-10 (collected on December 15).

No strains were isolated from samples: 1-5 and 11-15 (rainy); 21-25, 31-35 and 36-40 (dry); 46-50 and 51-55 (rainy).

R – Resistant

I – Intermediate

S - Sensible

AMR - antimicrobial resistance

IRA - Strain resistance index to antibiotic

IRAp - Strain resistance index to antibiotics

MAR – Multiple resistance index per collection site

Antibiotics: Amikacin 30 µg (AMI), Ampicillin 10 µg (AMP), Ampicillin + Sulbactam 20 µg (ASB), Aztreonam 30 µg (ATM), Cefazolin 30 µg (CFZ), Cefepime 30 µg (CPM), Cefotaxime 30 µg (CTX), Cefoxitin 30 µg (CFO), Ceftazidine 30 µg (CAZ), Ceftriaxone 30 µg (CRO), Ciprofloxacin 5 µg (CIP), Chloramphenicol 25 µg (CHL), Cotrimoxazole or Trimethoprim-Sulfamethoxazole 25 µg (SUT), Gentamicin 10 µg (GEN), Imipenem 10 µg (IPM), Levofloxacin 5 µg (LVX), Meropenem 10 µg (MER), Nitrofurantoin 300 µg (NIT), Piperacillin + Tazobactam 110 µg (PPT) and Tetracycline 30 µg (TET)

# **Table S8**

Table S8. Resistance profile of E. coli strains to one or more antibiotics among 20 tested antibiotics on the Beach 2. Index IRA (resistance index to antibiotic), Index IRAp (resistance index to antibiotics per beach), Index MAR (multiple antibiotic resistance per beach).

| **Samples** | 16-JAN | 16-MAR | **Frequency R (n=2)** | **Frequency I (n=2)** | **Frequency S (n=2)** | **IRA = N° R/n (n=2)** |
| --- | --- | --- | --- | --- | --- | --- |
|  | 11-15 | 21-25 |  |  |  |  |
| Tested antibiotics | rainy | dry |  |  |  |  |
| AMI | S | S | 0 | 0 | 2 | 0.0 |
| AMP | S | R | 1 | 0 | 1 | 0,5 |
| ASB | S | R | 1 | 0 | 1 | 0,5 |
| ATM | S | R | 1 | 0 | 1 | 0,5 |
| CFZ | S | R | 1 | 0 | 1 | 0,5 |
| CPM | S | R | 1 | 0 | 1 | 0,5 |
| CTX | S | R | 1 | 0 | 1 | 0,5 |
| CFO | S | R | 1 | 0 | 1 | 0,5 |
| CAZ | S | R | 1 | 0 | 1 | 0,5 |
| CRO | S | R | 1 | 0 | 1 | 0,5 |
| CIP | S | S | 0 | 0 | 2 | 0.0 |
| CHL | S | R | 1 | 0 | 1 | 0,5 |
| SUT | S | I | 0 | 1 | 1 | 0.0 |
| GEN | S | S | 0 | 0 | 2 | 0.0 |
| IPM | S | R | 1 | 0 | 1 | 0,5 |
| LVX | S | R | 1 | 0 | 1 | 0,5 |
| MER | S | S | 0 | 0 | 2 | 0.0 |
| NIT | S | S | 0 | 0 | 2 | 0.0 |
| PPT | S | I | 0 | 0 | 2 | 0.0 |
| TET | S | I | 0 | 1 | 1 | 0.0 |
| **Antibiotic resistance per point (drug Class)** | **0** | **12 (6)** | IRAp for beach = n with strain resistance  N° Samples  IRAp for beach = 1  11  **IRAp for beach** = **0.091** | | | |
| **Antibiotic intermediate per point** | **0** | **3** |  |  |  |  |
| **IRAp for sample =** N° AMR/∑ antibiotics = ∑R/20 | **0.00** | **0.60** |  |  |  |  |
| **Index (I) = N° Antibiotic intermediate / total antibiotics = n°I/20** | **0.00** | **0.15** |  |  |  |  |
| **MAR Index** = N° AMR/(n with strain resistance x ∑ antibiotics) = ∑R /(1*20) | **0.60** | |  |  |  |  |

Analysis was not performed on samples 6-10 (collected on December 15).

No strains were isolated from samples: 1-5 (rainy); 16-20, 26-30, 31-35, 36-40 and 41-45 (dry); 46-50, 51-55 and 56-60 (rainy).

R – Resistant

I – Intermediate

S - Sensible

AMR - antimicrobial resistance

IRA - Strain resistance index to antibiotic

IRAp - Strain resistance index to antibiotics

MAR – Multiple resistance index per collection site

Antibiotics: Amikacin 30 µg (AMI), Ampicillin 10 µg (AMP), Ampicillin + Sulbactam 20 µg (ASB), Aztreonam 30 µg (ATM), Cefazolin 30 µg (CFZ), Cefepime 30 µg (CPM), Cefotaxime 30 µg (CTX), Cefoxitin 30 µg (CFO), Ceftazidine 30 µg (CAZ), Ceftriaxone 30 µg (CRO), Ciprofloxacin 5 µg (CIP), Chloramphenicol 25 µg (CHL), Cotrimoxazole or Trimethoprim-Sulfamethoxazole 25 µg (SUT), Gentamicin 10 µg (GEN), Imipenem 10 µg (IPM), Levofloxacin 5 µg (LVX), Meropenem 10 µg (MER), Nitrofurantoin 300 µg (NIT), Piperacillin + Tazobactam 110 µg (PPT) and Tetracycline 30 µg (TET)

# **Table S9**

Table S9. Resistance profile of E. coli strains to one or more antibiotics among 20 tested antibiotics on the Beach 3. Index IRA (resistance index to antibiotic), Index IRAp (resistance index to antibiotics per beach), Index MAR (multiple antibiotic resistance per beach).

| **Samples** | **16-NOV** | **Frequency R (n=1)** | **Frequency I (n=1)** | **Frequency S (n=1)** | **IRA = N° R/n (n=1)** |
| --- | --- | --- | --- | --- | --- |
|  | **56 - 60** |  |  |  |  |
| Tested antibiotics | rainy |  |  |  |  |
| AMI | S | 0 | 0 | 1 | 0.0 |
| AMP | S | 0 | 0 | 1 | 0.0 |
| ASB | S | 0 | 0 | 1 | 0.0 |
| ATM | S | 0 | 0 | 1 | 0.0 |
| CFZ | S | 0 | 0 | 1 | 0.0 |
| CPM | S | 0 | 0 | 1 | 0.0 |
| CTX | S | 0 | 0 | 1 | 0.0 |
| CFO | S | 0 | 0 | 1 | 0.0 |
| CAZ | S | 0 | 0 | 1 | 0.0 |
| CRO | S | 0 | 0 | 1 | 0.0 |
| CIP | S | 0 | 0 | 1 | 0.0 |
| CHL | S | 0 | 0 | 1 | 0.0 |
| SUT | S | 0 | 0 | 1 | 0.0 |
| GEN | S | 0 | 0 | 1 | 0.0 |
| IPM | **R** | 1 | 0 | 0 | 1.0 |
| LVX | S | 0 | 0 | 1 | 0.0 |
| MER | S | 0 | 0 | 1 | 0.0 |
| NIT | S | 0 | 0 | 1 | 0.0 |
| PPT | S | 0 | 0 | 1 | 0.0 |
| TET | **S** | 0 | 0 | 1 | 0.0 |
| **Antibiotic resistance per point (drug Class)** | **1 (1)** | IRAp for beach = n with strain resistance  N° Samples  IRAp for beach = 1  11  **IRAp for beach** = **0.091** | | | |
| **Antibiotic intermediate per point** | **0** |  |  |  |  |
| **IRAp for sample =** N° AMR/∑ antibiotics = ∑R/20 | **0.05** |  |  |  |  |
| **Index (I) = N° Antibiotic intermediate / total antibiotics = n°I/20** | **0.00** |  |  |  |  |
| **MAR Index** = N° AMR/(n with strain resistance x ∑ antibiotics) = ∑R /(1*20) | **0.05** |  |  |  |  |

Analysis was not performed on samples 6-10 (collected on December 15).

No strains were isolated from samples: 1-5 and 11-15 (rainy); 16-20, 21-25, 26-30, 31-35, 36-40 and 41-45 (dry); 46-50 and 51-55(rainy).

R – Resistant

I – Intermediate

S - Sensible

AMR - antimicrobial resistance

IRA - Strain resistance index to antibiotic

IRAp - Strain resistance index to antibiotics

MAR – Multiple resistance index per collection site

Antibiotics: Amikacin 30 µg (AMI), Ampicillin 10 µg (AMP), Ampicillin + Sulbactam 20 µg (ASB), Aztreonam 30 µg (ATM), Cefazolin 30 µg (CFZ), Cefepime 30 µg (CPM), Cefotaxime 30 µg (CTX), Cefoxitin 30 µg (CFO), Ceftazidine 30 µg (CAZ), Ceftriaxone 30 µg (CRO), Ciprofloxacin 5 µg (CIP), Chloramphenicol 25 µg (CHL), Cotrimoxazole or Trimethoprim-Sulfamethoxazole 25 µg (SUT), Gentamicin 10 µg (GEN), Imipenem 10 µg (IPM), Levofloxacin 5 µg (LVX), Meropenem 10 µg (MER), Nitrofurantoin 300 µg (NIT), Piperacillin + Tazobactam 110 µg (PPT) and Tetracycline 30 µg (TET)

# **Table S10**

Table S10. Resistance profile of E. coli strains to one or more antibiotics among 20 tested antibiotics on the Beach 4. Index IRA (resistance index to antibiotic), Index IRAp (resistance index to antibiotics per beach), Index MAR (multiple antibiotic resistance per beach).

| **Samples** | **15-NOV** | **Frequency R (n=1)** | **Frequency I (n=1)** | **Frequency S (n=1)** | **IRA = N° R/n (n=1)** |
| --- | --- | --- | --- | --- | --- |
|  | **1 - 5** |  |  |  |  |
| Tested antibiotics | rainy |  |  |  |  |
| AMI | S | 0 | 0 | 1 | 0.0 |
| AMP | **R** | 1 | 0 | 0 | 1.0 |
| ASB | **I** | 0 | 1 | 0 | 0.0 |
| ATM | S | 0 | 0 | 1 | 0.0 |
| CFZ | **R** | 1 | 0 | 0 | 1.0 |
| COM | S | 0 | 0 | 1 | 0.0 |
| CTX | S | 0 | 0 | 1 | 0.0 |
| CFO | S | 0 | 0 | 1 | 0.0 |
| CAZ | S | 0 | 0 | 1 | 0.0 |
| CRO | S | 0 | 0 | 1 | 0.0 |
| CIP | S | 0 | 0 | 1 | 0.0 |
| CHL | S | 0 | 0 | 1 | 0.0 |
| SUT | S | 0 | 0 | 1 | 0.0 |
| GEN | S | 0 | 0 | 1 | 0.0 |
| IPM | S | 0 | 0 | 1 | 0.0 |
| LVX | S | 0 | 0 | 1 | 0.0 |
| MER | S | 0 | 0 | 1 | 0.0 |
| NIT | S | 0 | 0 | 1 | 0.0 |
| PPT | **I** | 0 | 1 | 0 | 0.0 |
| TET | **R** | 1 | 0 | 0 | 1.0 |
| **Antibiotic resistance per point (drug Class)** | **3 (3)** | IRAp for beach = n with strain resistance  N° Samples  IRAp for beach = 1  11  **IRAp for beach** = **0.091** | | | |
| **Antibiotic intermediate per point** | **2** |  |  |  |  |
| **IRAp for sample =** N° AMR/∑ antibiotics = ∑R/20 | **0.15** |  |  |  |  |
| **Index (I) = N° Antibiotic intermediate / total antibiotics = n°I/20** | **0.10** |  |  |  |  |
| **MAR Index** = N° AMR/(n with strain resistance x ∑ antibiotics) = ∑R /(10*20) | **0.15** |  |  |  |  |

Analysis was not performed on samples 6-10 (collected on December 15).

No strains were isolated from samples: 11-15 (rainy); 16-20, 21-25, 26-30, 31-35, 36-40 and 41-45 (dry); 46-50, 51-55 and 56-60 (rainy).

R – Resistant

I – Intermediate

S - Sensible

AMR - antimicrobial resistance

IRA - Strain resistance index to antibiotic

IRAp - Strain resistance index to antibiotics

MAR – Multiple resistance index per collection site

Antibiotics: Amikacin 30 µg (AMI), Ampicillin 10 µg (AMP), Ampicillin + Sulbactam 20 µg (ASB), Aztreonam 30 µg (ATM), Cefazolin 30 µg (CFZ), Cefepime 30 µg (CPM), Cefotaxime 30 µg (CTX), Cefoxitin 30 µg (CFO), Ceftazidine 30 µg (CAZ), Ceftriaxone 30 µg (CRO), Ciprofloxacin 5 µg (CIP), Chloramphenicol 25 µg (CHL), Cotrimoxazole or Trimethoprim-Sulfamethoxazole 25 µg (SUT), Gentamicin 10 µg (GEN), Imipenem 10 µg (IPM), Levofloxacin 5 µg (LVX), Meropenem 10 µg (MER), Nitrofurantoin 300 µg (NIT), Piperacillin + Tazobactam 110 µg (PPT) and Tetracycline 30 µg (TET)

# **Table S11**

Table S11. Resistance profile of E. coli strains to one or more antibiotics among 20 tested antibiotics on Beach 5. Index IRA (resistance index to antibiotic), Index IRAp (resistance index to antibiotics per beach), Index MAR (multiple antibiotic resistance per beach).

| **Samples** | **15-NOV** | **16-FEB** | **16-MAR** | **16-MAY** | **16-JUN** | **16-JUL** | **16-AUG** | **16-SEP** | **16-OCT** | **16-NOV** | **Frequency R (n=1)** | **Frequency I (n=1)** | **Frequency S (n=1)** | **IRA = N° R/n (n=1)** |
| --- | --- | --- | --- | --- | --- | --- | --- | --- | --- | --- | --- | --- | --- | --- |
|  | **1 - 5** | **16 - 20** | **21 - 25** | **26 - 30** | **31 - 35** | **36 - 40** | **41 - 45** | **46 - 50** | **51 - 55** | **56 - 60** |  |  |  |  |
| Tested antibiotics | rainy | dry | dry | dry | dry | dry | dry | rainy | rainy | rainy |  |  |  |  |
| AMI | S | S | S | S | S | S | S | S | S | S | 0 | 0 | 10 | 0.0 |
| AMP | **I** | S | **R** | **I** | S | S | S | **I** | S | S | 1 | 3 | 6 | 0.1 |
| ASB | S | S | **R** | S | S | S | S | S | S | S | 1 | 0 | 9 | 0.1 |
| ATM | S | S | **R** | S | S | S | S | S | S | S | 1 | 0 | 9 | 0.1 |
| CFZ | **R** | **I** | **R** | **R** | S | **R** | **I** | S | **R** | S | 5 | 2 | 3 | 0.5 |
| CPM | S | S | **R** | **R** | S | S | S | S | S | S | 2 | 0 | 8 | 0.2 |
| CTX | **R** | S | **R** | S | S | S | S | S | S | S | 2 | 0 | 8 | 0.2 |
| CFO | S | S | **I** | S | S | S | S | S | **R** | S | 1 | 1 | 8 | 0.1 |
| CAZ | S | S | **R** | **R** | S | S | S | S | S | S | 2 | 0 | 8 | 0.2 |
| CRO | S | S | **R** | S | S | S | S | S | S | S | 1 | 0 | 9 | 0.1 |
| CIP | S | S | S | **I** | **I** | S | S | S | S | S | 0 | 2 | 8 | 0.0 |
| CHL | **R** | **I** | **R** | S | S | S | S | **I** | **I** | S | 2 | 3 | 5 | 0.2 |
| SUT | **I** | S | **I** | S | S | S | S | S | S | S | 0 | 2 | 8 | 0.0 |
| GEN | S | S | S | S | S | S | S | S | S | S | 0 | 0 | 10 | 0.0 |
| IPM | S | S | S | S | **R** | **I** | **R** | **R** | **R** | **R** | 5 | 1 | 4 | 0.5 |
| LVX | S | S | S | S | S | S | S | S | S | S | 0 | 0 | 10 | 0.0 |
| MER | S | S | S | S | S | S | S | S | S | S | 0 | 0 | 10 | 0.0 |
| NIT | S | S | S | S | S | S | S | S | S | S | 0 | 0 | 10 | 0.0 |
| PPT | **I** | S | **I** | S | S | S | S | S | S | S | 0 | 0 | 10 | 0.0 |
| TET | S | S | S | S | S | S | S | S | S | **R** | 1 | 0 | 9 | 0.1 |
| **Antibiotic resistance per point (drug Class)** | **3 (2)** | **0** | **9 (4)** | **3 (1)** | **1 (1)** | **1 (1)** | **1 (1)** | **1 (1)** | **3 (2)** | **2 (2)** | IRAp for beach = n with strain resistance  N° Samples  IRAp for beach = 9  11  **IRAp for beach** = **0.818** | | | |
| **Antibiotic intermediate per point** | **3** | **2** | **3** | **2** | **1** | **1** | **1** | **2** | **1** | **0** |  |  |  |  |
| **IRAp for sample =** N° AMR/∑ antibiotics = ∑R/20 | **0.15** | **0.00** | **0.45** | **0.15** | **0.05** | **0.05** | **0.05** | **0.05** | **0.15** | **0.10** |  |  |  |  |
| **Index (I) = N° Antibiotic intermediate / total antibiotics = n°I/20** | **0.15** | **0.10** | **0.15** | **0.10** | **0.05** | **0.05** | **0.05** | **0.10** | **0.05** | **0.00** |  |  |  |  |
| **MAR Index** = N° AMR/(n with strain resistance x ∑antibiotics) = ∑R /(9*20) | | | | | | | **0.13** | | |  |  |  |  |  |

Analysis was not performed on samples 6-10 (collected on December 15).

No strains were isolated from samples: 11-15 (rainy).

R – Resistant

I – Intermediate

S - Sensible

AMR - antimicrobial resistance

IRA - Strain resistance index to antibiotic

IRAp - Strain resistance index to antibiotics

MAR – Multiple resistance index per collection site

Antibiotics: Amikacin 30 µg (AMI), Ampicillin 10 µg (AMP), Ampicillin + Sulbactam 20 µg (ASB), Aztreonam 30 µg (ATM), Cefazolin 30 µg (CFZ), Cefepime 30 µg (CPM), Cefotaxime 30 µg (CTX), Cefoxitin 30 µg (CFO), Ceftazidine 30 µg (CAZ), Ceftriaxone 30 µg (CRO), Ciprofloxacin 5 µg (CIP), Chloramphenicol 25 µg (CHL), Cotrimoxazole or Trimethoprim-Sulfamethoxazole 25 µg (SUT), Gentamicin 10 µg (GEN), Imipenem 10 µg (IPM), Levofloxacin 5 µg (LVX), Meropenem 10 µg (MER), Nitrofurantoin 300 µg (NIT), Piperacillin + Tazobactam 110 µg (PPT) and Tetracycline 30 µg (TET)

# **Table S12**

Table S12. The presented antimicrobials are grouped into the following pharmacological classes (WHO, 2023).

| **Pharmacological classes** | **Antibiotics and concentration used in this study** |
| --- | --- |
| Aminoglycosides | Amikacin 30 µg (AMI), Gentamicin 10 µg (GEN) |
| Penicillins and Combinations | Ampicillin 10 µg (AMP), Ampicillin + Sulbactam 20 µg (ASB), Piperacillin + Tazobactam 110 µg (PPT) |
| Cephalosporins | Cefazolin 30 µg (CFZ), Cefoxitin 30 µg (CFO), Cefotaxime 30 µg (CTX), Ceftazidine 30 µg (CAZ), Ceftriaxone 30 µg (CRO), Cefepime 30 µg (CPM) |
| Carbapenems | Imipenem 10 µg (IPM), Meropenem 10 µg (MER) |
| Monobactams | Aztreonam 30 µg (ATM) |
| Fluoroquinolones | Ciprofloxacin 5 µg (CIP), Levofloxacin 5 µg (LVX) |
| Phenicols | Chloramphenicol 25 µg (CHL) |
| Sulfonamides / Antifolates | Cotrimoxazole 25 µg (SUT) |
| Tetracyclines | Tetracycline 30 µg (TET) |
| Nitrofurans | Nitrofurantoin 300 µg (NIT) |

# **Table S13**

Table S13. Antimicrobial susceptibility profile of *E. coli* - positive samples.

| **Antimicrobials tested** |  | **Antimicrobial susceptibility profile** | | | |
| --- | --- | --- | --- | --- | --- |
|  | Abbreviation | ***Resistant n (%)*** | ***Intermediate n (%)*** | ***Sensitive n (%)*** | **IRA (%) = N°R/_total antibiotic tested ⇒_ IRA = N° R/20** |
| Amikacin 30 µg | AMI | 0.0% | 0.0% | 18 (100%) | 0.00 |
| Ampicillin 10 µg | AMP | 3 (16.7%) | 4 (22.2%) | 11 (61.1%) | 0.15 |
| Ampicillin + Sulbactam 20 µg | ASB | 2 (11.1%) | 1 (5.6%) | 15 (83.3%) | 0.10 |
| Aztreonam 30 µg | ATM | 2 (11.1%) | 0.0% | 16 (88.9%) | 0.10 |
| Cefazolin 30 µg | CFZ | 7 (38.9%) | 4 (22.2%) | 7 (38.9%) | **0.35** |
| Cefepime 30 µg | CPM | 3 (16.7%) | 1 (5.6%) | 14 (77.8%) | 0.15 |
| Cefotaxime 30 µg | CTX | 3 (16.7%) | 0.0% | 15 (83.3%) | 0.15 |
| Cefoxitin 30 µg | CFO | 2 (11.1%) | 1 (5.6%) | 15 (83.3%) | 0.10 |
| Ceftazidine 30 µg | CAZ | 3 (16.7%) | 0.0% | 15 (83.3%) | 0.15 |
| Ceftriaxone 30 µg | CRO | 2 (11.1%) | 0.0% | 16 (88.9%) | 0.10 |
| Ciprofloxacin 5 µg | CIP | 0.0% | 2 (11.1%) | 16 (88.9%) | 0.00 |
| Chloramphenicol 25 µg | CHL | 4 (22.2%) | 4 (22.2%) | 10 (55.6%) | **0.20** |
| Cotrimoxazol or Trimethoprim-Sulfamethoxazole 25 µg | SUT | 0.0% | 3 (16.7%) | 15 (83.3%) | 0.00 |
| Gentamicin 10 µg | GEN | 0.0% | 0.0% | 18 (100%) | 0.00 |
| Imipenem 10 µg | IPM | 9 (50.0%) | 1 (5.6%) | 8 (44.4%) | **0.45** |
| Levofloxacin 5 µg | LVX | 1 (5.6%) | 0.0% | 17 (94.4%) | 0.05 |
| Meropenem 10 µg | MER | 0.0% | 0.0% | 18 (100%) | 0.00 |
| Nitrofurantoin 300 µg | NIT | 0.0% | 0.0% | 18 (100%) | 0.00 |
| Piperacillin+Tazobactam 110 µg | PPT | 0.0% | 5 (27,8%) | 13 (72.2%) | 0.00 |
| Tetracycline 30 µg | TET | 3 (16.7%) | 1 (5.6%) | 14 (77.8%) | 0.15 |

# **Figure S1**

A

IRA - resistance index per antibiotic

|  |  |
| --- | --- |
| AMI | 0 |
| AMP | 0.15 |
| ASB | 0.1 |
| ATM | 0.1 |
| CFZ | 0.35 |
| CPM | 0.15 |
| CTX | 0.15 |
| CFO | 0.1 |
| CAZ | 0.15 |
| CRO | 0.1 |
| CIP | 0 |
| CLO | 0.2 |
| SUT | 0 |
| GEN | 0 |
| IPM | 0.45 |
| LVX | 0.05 |
| MER | 0 |
| NIT | 0 |
| PPT | 0 |
| TET | 0.15 |

B

Antimicrobial Classes

|  |  |
| --- | --- |
| Beach 1 (16-FEB) | 1 |
| Beach 1 (16-MAY) | 0 |
| Beach 1 (16-AUG) | 1 |
| Beach 1 (16-NOV) | 2 |
| Beach 2 (16-JAN) | 0 |
| Beach 2 (16-MAR) | 6 |
| Beach 3 (16-NOV) | 1 |
| Beach 4 (15-NOV) | 3 |
| Beach 5 (15-NOV) | 2 |
| Beach 5 (16-FEB) | 0 |
| Beach 5 (16-MAR) | 4 |
| Beach 5 (16-MAY) | 1 |
| Beach 5 (16-JUN) | 1 |
| Beach 5 (16-JUL) | 1 |
| Beach 5 (16-AUG) | 1 |
| Beach 5 (16-SEP) | 1 |
| Beach 5 (16-OCT) | 2 |
| Beach 5 (16-NOV) | 2 |

Figure S1. (A) Comparison of antibiotic resistance among *Escherichia coli* isolates from some of the beaches. (B) Antimicrobial classes resistance among *E. coli* isolates from each beach.

# **Table S14**

Table S14. Factor coordinates of the variables, based on correlations.

| **Variable** | **Factor 1** | **Factor 2** | **Factor 3** | **Factor 4** |  |
| --- | --- | --- | --- | --- | --- |
| DO | -0,353567 | 0,909788 | -0,158726 | -0,1486 |  |
| Temperature | -0,829149 | -0,424148 | 0,209596 | -0,297793 |  |
| Resistance | -0,903163 | 0,074366 | 0,140099 | 0,398923 |  |
| Sum of Strains | -0,95166 | 0,245241 | 0,170531 | -0,071553 |  |
| Coliforms | -0,779328 | -0,347147 | -0,521585 | 0,009312 |  |

# **Table S15**

Table S15. Eigenvalues of correlation matrix, and related statistics. Active variables Only.

| **Value number** | **Eigenvalue** | **% Total variance** | **Cumulative Eigenvalue** | **Cumulative %** |
| --- | --- | --- | --- | --- |
| 1 | 3,141207 | 62,82415 | 3,141207 | 62,8241 |
| 2 | 1,1938 | 23,876 | 4,335007 | 86,7001 |
| 3 | 0,389884 | 7,79768 | 4,724891 | 94,4978 |
| 4 | 0,275109 | 5,50217 | 5 | 100 |

# **References**

- CONAMA. CONSELHO NACIONAL DO MEIO AMBIENTE (2000). Resolução Conama nº 274, de 29 de novembro de 2000. Define os critérios de balneabilidade em águas brasileiras. Brazil. http://www2.mma.gov.br/port/conama/legiabre.cfm?codlegi=272 (in portuguese).
- CONAMA. CONSELHO NACIONAL DO MEIO AMBIENTE (2005). Resolução Conama nº 357, de 18 de março de 2005. Dispõe sobre a classificação dos corpos de água e diretrizes ambientais para o seu enquadramento, bem como estabelece as condições e padrões de lançamento de efluentes, e dá outras providências. Brazil. http://www2.mma.gov.br/port/conama/legiabre.cfm?codlegi=459 (in portuguese).
- WHO - World Health Organization (2023). AWaRe classification of antibiotics for evaluation and monitoring of use, 2023. World Health Organization. Retrieved from https://www.who.int/publications/i/item/WHO-MHP-HPS-EML-2023.04. Accessed 30 August 2026.
